# Cleavage pattern, morula compaction and blastocyst morphology as determinants of live birth after single blastocyst transfer

**DOI:** 10.3389/frph.2026.1822869

**Published:** 2026-04-15

**Authors:** Emma Adolfsson, Amanda Stenberg, Juliane Baumgart

**Affiliations:** 1 Department of Obstetrics and Gynecology, Faculty of Medicine and Health, Örebro University, Örebro, Sweden; 2 Department of Obstetrics and Gynecology, Örebro University Hospital, Örebro, Sweden

**Keywords:** assisted reproductive technology, cleavage pattern, embryo development, embryo selection, live birth predictors, morula, morula compaction, time-lapse imaging

## Abstract

**Introduction:**

This retrospective time lapse study evaluated 3,103 transferred autologous blastocysts to determine how early division patterns, morula compaction behavior and blastocyst quality influence clinical outcomes.

**Methods:**

Embryos were categorised by cleavage pattern (normal or abnormal), degree of morula compaction (full or partial), and blastocyst quality (top, good or low).

**Result:**

Most transferred blastocysts, 92.5%, originated from normally dividing embryos, of which 63.8% developed into fully compacted morulas. In unadjusted analyses, fully compacted morulas resulted in higher pregnancy, clinical pregnancy and live birth rates than partially compacted morulas across all morphology categories. Embryos with abnormal cleavage constituted 7.5% of the cohort, developed almost exclusively into partial morulas, and showed reduced reproductive potential, with lower pregnancy and clinical pregnancy rates compared with normally dividing embryos, and lower live birth rates compared with partial morulas originating from normal cleavage. The highest live birth rate (38.9%) was observed for top quality blastocysts originating from normally cleaving, fully compacted morulas. In multivariable models adjusting for maternal age and blastocyst developmental day, blastocyst morphology and blastocyst age were the strongest independent predictors of clinical outcome, while maternal age showed a consistent negative association. Abnormal cleavage remained associated with reduced pregnancy and clinical pregnancy rates, although this effect did not persist for live birth, and compaction pattern did not retain significance after adjustment.

**Discussion:**

Overall, early developmental behavior, particularly cleavage pattern and morula compaction, aligns with downstream morphology to shape embryo competence, while blastocyst morphology and blastocyst developmental day remain the primary determinants of live birth after single blastocyst transfer.

## 1 Introduction

Embryo selection is the process when embryologists identify embryos with high implantation ability for transfer or cryopreservation. Equally important is to identify and deselect embryos without the ability to give successful outcomes to shorten time to pregnancy (TPP), reduce stress for patient/s and risk of drop-out after transfers. Selecting and deselecting embryos using non-invasive methods remains challenging. Embryos may appear morphologically normal and of high quality yet still be aneuploid (1). In many countries PGT-A is not allowed, and for others it is not affordable. Improvements in non-invasive embryo assessment and a deeper understanding of critical developmental milestones may therefore improve assisted reproduction outcomes without the added cost of genetic testing.

Time-lapse imaging (TLI) catches all aspects of embryo development and allows for uninterrupted culture with continuous monitoring. TLI has revealed important information about cell-division kinetics and division patterns, and its use has been incorporated into clinical guidelines (2). Importantly, TLI captures critical milestones not observable in conventional incubators with fixed time observations. One such event is the first mitosis which occurs at night, approximately at 27 h post insemination. Errors in the first mitosis can appear as nuclear error phenotypes in the two-cell embryo which is correlated with poorer blastocyst formation rate (3). Another error is direct unequal cleavage (DUC), defined as division of a single blastomere into three or more cells. The earlier DUC occurs, the greater the damage. DUC1–3 (Supplementary Video S1) occurs in the first mitosis and results in the cleavage of the fertilized zygote into three mitotic daughter cells (tripolar mitosis). These three cells are genetically abnormal (4, 5) and ESHRE advise against their clinical use.

Direct cleavage can also occur in the second mitosis, resulting in a five-cell embryo (DUC2–5), or more rarely, to a six-cell embryo (DUC2–6) if both blastomeres divide abnormally. In DUC2–5 (Supplementary Video S2), genetically normal cells can still progress to form a morula and blastocyst, whereas abnormal cells typically arrest (4, 6, 7). In contrast, DUC2–6 yield only abnormal daughter cells similar to DUC1–3, and embryos with this pattern should also be avoided for transfer.

Another abnormal cell division pattern is rapid cleavage (RaC) (Supplementary Video S3) where one of the two cells resulting from the first mitosis divide again within less than 5 h, producing a cell stage size-inappropriate three-cell embryo. This shortened cell cycle does not allow sufficient time for chromosomal duplication and results in one normal and two abnormal cells (7). Like DUC2–5 embryos, RaC embryos may contain both normal and abnormal cells and can progress to partial compacted morulas and blastocysts.

All these abnormal division patterns are associated with reduced blastocyst formation (8, 9), lower blastocyst quality (10, 11), and decreased implantation rates when transferred at the cleavage stage (12–14). Culturing embryos to the blastocyst stage may allow embryos with aberrations in the genetic constitution to self-arrest, therefore giving embryologists more information and improving embryo selection. Furthermore, using TLI with blastocyst culture also captures the transition from cleavage stage to blastocyst, i.e., the morula stage normally occurring on day 4 of culture.

The morula is defined as the cell mass formed by cleavage of the ovum before blastocyst formation. Tight junctions are formed between the cells, leading to the visual disappearance of cell boundaries. Two patterns of compaction are observed: fully compacted morulas (FCM) (Supplementary Video S4) incorporating all mitotic cells, and partially compacted morulas (PCM) (Supplementary Video S5) where at least one mitotic cell remains excluded (1, 15–17). PCM have been associated with reduced reproductive outcomes (17–21).

Recently, a retrospective time-lapse study on 835 blastocysts reported that embryos with abnormal cleavage patterns (DUC1, RaC, DUC2) did not form FCM, whereas∼60% of embryos with normal cleavage patterns did (1). When biopsied, the aneuploidy rates were the same, and the authors suggested that exclusion of cells and formation of PCM could be a self-correction mechanism after abnormal cell divisions. This has also been hypothesized by others (15–17). Interestingly, another publication from 2024 by Lee at al. reported that abnormal cleavage patterns up until day 3 did not compromise clinical outcomes if the embryo was successfully cultured to the blastocyst and transferred after full blastulation, although the embryós ability to reach blastocyst stage was compromised (22). Furthermore, Parriego et al. reported from their retrospective time-lapse study on 872 blastocysts that if they resulted from PCM they had the same reproductive outcomes as blastocysts resulting from FCM. They suggested that blastocysts from PCM were not defined by their early mistakes (23).

This study investigates the frequency of abnormal cell divisions in transferred blastocysts, combined with morula compaction patterns and subsequent morphological quality, using the power of TLI to capture key milestones in embryo development. All included blastocysts were transferred with known clinical outcomes (home urine pregnancy tests, early ultrasounds with fetal heartbeat detection and live birth reports).

## 2 Materials and methods

This is a retrospective cohort study, carried out in a single ART clinic, the Reproductive Medicine Centre at the University Hospital of Örebro, Sweden.

Information was collected on 3,103 blastocysts from 1,740 ART cycles in 1,456 couples treated between 2012 and 2025 all with known clinical outcomes. The inclusion criteria were a) embryos obtained after IVF/ICSI and cultured in uninterrupted time-lapse incubators, b) fresh single embryo transfer on day 5, or frozen single embryo transfer with a vitrified/warmed day 5 or day 6 blastocyst, c) complete clinical outcome including urine pregnancy test results, early vaginal ultrasound with report on fetal heartbeat and number of sacs, and birth reports.

### 2.1 Human right statement and informed consent

All procedures were in accordance with the ethical standards of the responsible committee on human experimentation (institutional and national) and with the Helsinki Declaration of 1964 and its later amendments. This research study was conducted retrospectively from data obtained for clinical purposes. Patients had the option to consent to participating in quality assurance and/or methodological improvements prior to their assisted reproduction treatment. Only patients actively consenting to research and/or evaluation of treatments were included in this retrospective study. This approach was deemed satisfactory to the ethical committee, and no additional consent to participate in this retrospective study was required.

The study was approved by the Swedish Ethical Review Authority, ID 023-01153-0.

### 2.2 Data collection, categorization and analysis

For each blastocyst, a retrospective re-analysis was performed by a senior embryologist who reviewed all available time-lapse images from the EmbryoScope database (Vitrolife, Gothenburg, Sweden) with specific focus on the events relevant to this study. All observations were made blinded to the clinical outcomes. Each blastocyst was assessed for abnormalities in the first and/or second mitotic division. Embryos dividing in the expected sequence from one cell to two cells to four cells in two appropriately timely rounds of mitosis were classified as Normal Cleavage (NC). Embryos dividing directly from one cell to three cells in the first mitosis were categorized as DUC1–3 (24), and embryos with rapid cleavage from two to three cells within five hours were categorized as RaC (t3-t2 ≤5 h) (12, 25). Embryos with a cleavage pattern from two cells to five or more cells in the second mitosis were categorized as DUC2–5 or DUC2–6.

In all blastocysts, the transition from cleavage-stage to the blastocyst was examined from initiation of compaction to the onset of cavitation. Each morula was classified as fully compacted (FCM) or partially compacted (PCM) (1) (23). Finally, the resulting blastocyst was graded using the Gardner scoring system based on expansion, inner cell mass (ICM) and trophectoderm (TE) quality (26). Based on the Gardner scoring system, the blastocyst was categorized as Top Quality Blastocyst (TQB), Good Quality Blastocyst (GQB) or Low Quality Blastocyst (LQB). The TQB group included blastocysts with an A in either ICM, TE or both, and expansion grade ≥4 on day 5 and ≥5 on day 6. The GQB group included 3AA, 3AB, 3BA on day 5, and all blastocysts with BB and expansion grade ≥3 on both day 5 and day 6. The LQB group included all blastocysts with C in either ICM and/or TE, and all blastocysts with expansion grade 0, 1 or 2.

### 2.3 Controlled ovarian stimulation, oocyte retrieval and embryo culture

Embryos were obtained by controlled hormone ovarian stimulation monitored by ultrasounds. Stimulation was done either with an agonist regime (*n* = 90, 11.8%) or with an antagonist regime (*n* = 597, 88.2%) using recombinant or urine derived FSH. When >3 follicles reached 17 mm, a trigger was given to resume meiosis, either hCG or GnRH agonist trigger. Oocytes were collected 36 h post triggering using transvaginal aspiration after paracervical blockade and using intravenous morphine as pain relief. Oocytes were fertilized using either conventional IVF or ICSI and cultured in EmbryoScope or EmbryoScope+ in +37°C, 6% CO_2_ and 5% O_2_. Images were taken in several focal plans every 15 min and stored on a local server. On day 5 of culture, the best available blastocyst was transferred to the woman, or, if a freeze-all strategi was planned, all high-quality blastocysts were vitrified either on day 5, or if not ready, on day 6 using Rapid-I with RapidVit Blast (Vitrolife, Göteborg, Sweden). Vitrified blastocysts were individually warmed and transferred in a frozen embryo replacement cycle, either in natural cycle or in hormone stimulated cycle.

A home urine pregnancy test was performed 13 days after transfer. If the result was positive (HCG > 25 ng/mL), a vaginal ultrasound was performed in pregnancy week 8–9 to confirm the location of the pregnancy, the number of amniotic sacs, the number of fetuses, and fetal viability. After delivery, the patients reported pregnancy outcomes, birthweight, sex, and neonatal health.

### 2.4 Statistical analysis

Division patterns (normal or abnormal first mitosis and second mitosis), morula compaction (full/partial) and morphological quality were evaluated and correlated with clinical outcomes. Clinical outcomes collected included the number of positive pregnancy tests to calculate Pregnancy Rate (PR); number of observed fetal heartbeats in vaginal scans to calculate Clinical Pregnancy Rate (CPR) and number of transfers resulting in live birth, to calculate Live Birth Rate (LBR). Ectopic pregnancies (*n* = 8) and stillborn (*n* = 6) were excluded in the analysis of CPR and LBR, respectively. Categorical variables were compared using Fisher's exact test for 2 × 2 comparisons, or chi-square test with *post hoc* analysis for comparisons between multiple groups. All statistical analyses were done in Rstudio (version 2025.05.01) using R (version 4.4.1). Differences were considered statistically significant at *p* < 0.05.

The association between embryo characteristics and clinical outcomes was evaluated using multivariable regression models. Three binary outcomes were analyzed separately: pregnancy rate (PR), clinical pregnancy rate (CPR), and live birth rate (LBR). For each outcome, we fitted multivariable generalized linear models (logit link) including the following predictors: blastocyst morphological grade (top, good, or low), blastocyst age at transfer (day 5 vs. day 6), cleavage pattern (normal vs. abnormal), degree of compaction (fully compacted vs. partial compaction), and maternal age at oocyte pick-up (continuous, per year). Top quality blastocysts, day 5 blastocysts, normal cleavage, and full compaction were used as reference categories.

Effect sizes were expressed as average marginal effects (AMEs), representing the adjusted absolute change in predicted probability (in percentage points) of the outcome associated with each predictor, averaged across all individuals in the dataset. For categorical predictors, AMEs quantify the difference in predicted probability compared with the reference level; for continuous predictors (maternal age), AMEs represent the change in predicted probability per one-unit increase.

AMEs were chosen instead of odds ratios because they provide directly interpretable, population-averaged effect estimates, which are well suited for binary clinical outcomes and can be compared across models. Ninety-five percent confidence intervals for AMEs were obtained using standard errors derived from the delta method.

## 3 Results

### 3.1 General data

This study evaluated 3,103 blastocysts from 1,740 oocyte retrievals in 1,456 patient couples using autologous gametes. Female age at oocyte retrieval ranged from 20 to 42 years (mean age 32 ± 3.5), and partner age ranged from 21 to 58 years (mean age 35 ± 4.2). The cumulative live birth rate per oocyte retrieval was 65.1%. All transfers were single blastocyst transfers.

The overall PR per transfer was 51.9%, the CPR was 43.9% and the LBR 33.8%. Day 5 blastocysts (*n* = 2,576) had significantly better outcomes than day 6 blastocysts (*n* = 527): PR 54.0% vs. 41.7%, *p* < .001, CPR 46.1% vs. 33.2%, *p* < .001 and LBR 33.6% vs. 25.4%, *p* < .001. Blastocysts originating from ICSI (*n* = 1,451) had slightly higher PR, CPR and LBR compared to blastocysts from conventional IVF (*n* = 1,652); PR 53.2% vs. 50.7% *p* = .125, CPR 45.5% vs. 42.5%, *p* < .05, and LBR 35.3% vs. 32.4%, *p* < .05.

### 3.2 Normal cell division patterns, morula compaction and outcome

Most of the transferred blastocysts (92.5%) divided as expected from one cell to two to four during the first two mitotic divisions. Of the 2,872 embryos, 63.8% (*n* = 1,832), developed into FCM and 36.2% (*n* = 1,040) developed into PCM. Most normally dividing embryos reached the blastocyst stage on day 5 (84.4%), while 15.6% reached it on day 6. Clinical outcomes differed significantly between the two morula groups with higher PR, CPR and LBR for FCM compared to PCM, see Table 1 for details.

**Table 1:** Clinical outcomes of normally divided blastocysts grouped on basis of their morula compaction pattern.

Morula pattern	Number (*n*)	Fraction (%)	PR (%)	CPR (%)	LBR (%)
Fully Compacted Morula	1,832	63.8	55	47.3	36.8
Partial Compacted Morula	1,040	36.2	49	40.8	30.5
*p*-value			<0.01	<0.001	<0.01

Data are shown as numbers and fractions, and Fisheŕs exact *t*-test was used to evaluate the differences between proportions.

PR, pregnancy rate; CPR, clinical pregnancy rate; LBR, live birth rate; all expressed per transfer.

**Figure 1:**
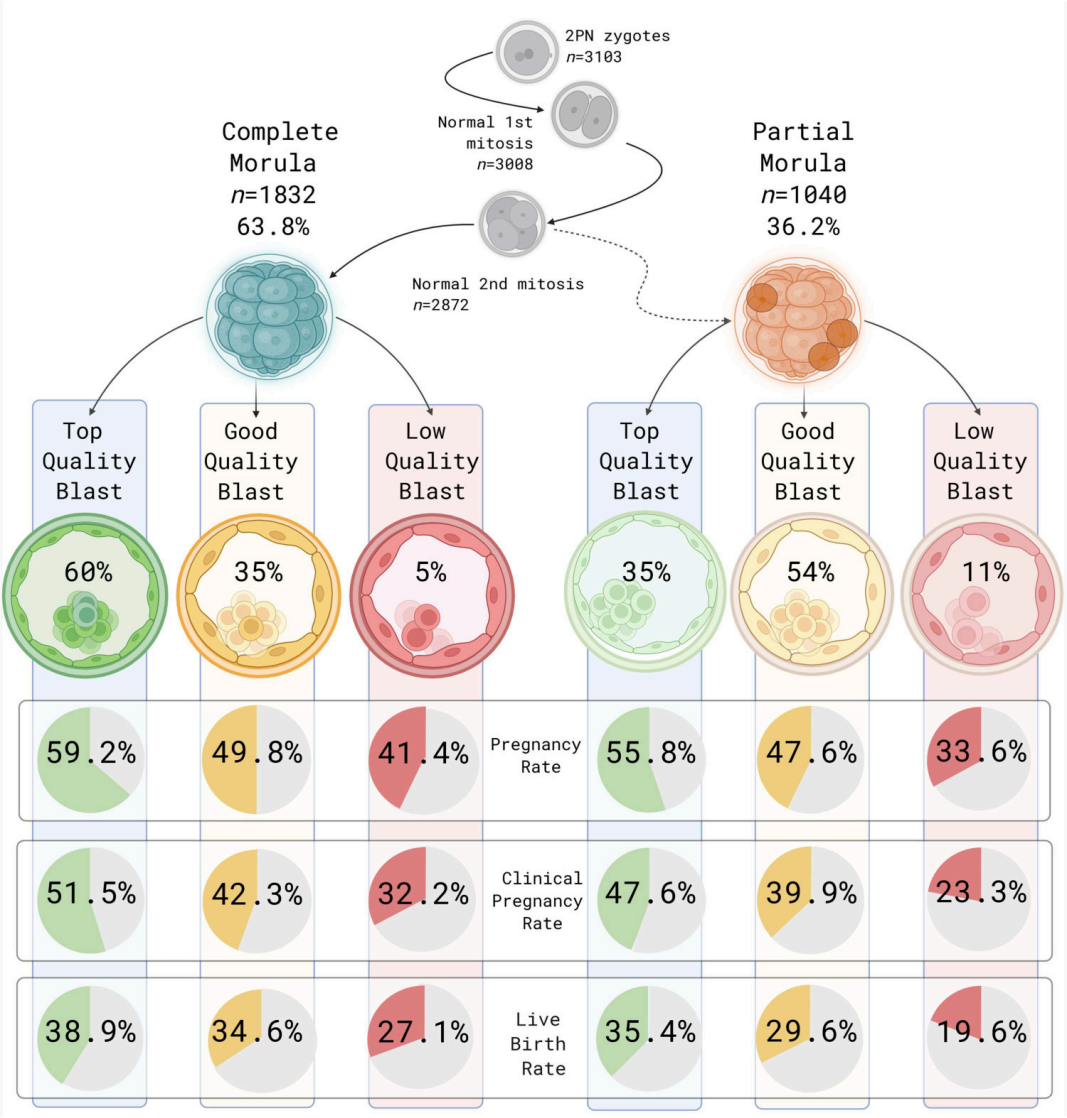
Distribution of morula formation pattern, blastocyst morphological quality and clinical outcomes of transferred blastocysts with normal cell divisions. Created in BioRender. Adolfsson, E. (2026) https://BioRender.com/zqn5xa0.

The FCM category developed mainly into TQB (60.0%), GQB (35.2%), and less commonly into LQB (4.8%). The PCM category developed into mainly GQB (54.2%), a lower proportion of TQB (34.8%) and a larger proportion of LQB (11.0%) compared with FCM, see Figure 1. Expressed the other way around, TQB (*n* = 1,454) originated to a significantly greater extent from FCM compared to PCM, 75.3% vs. 25.7%, *p* < .001. GQB (*n* = 1,201) originated in similar proportions from FCM (53.5%) and PCM (46.5%) and LQB (*n* = 201) also originated in equal fractions from FCM (43.3%) and PCM (56.7%).

Morphological quality had a significant impact on clinical outcomes. TQB transfers resulted in significantly higher PR, CPR and LBR compared to both GQB and LQB, see Table 2. The significant differences in clinical outcomes between fully and partially compacted morulas persisted when combining morula quality with subsequent blastocyst morphology quality, visualized in Figure 1. Interestingly, for all morphology grades, blastocysts originating from FCM resulted in higher PR, CPR and LBR compared to blastocysts originating from PCM, emphasizing that the morula compaction pattern influences clinical outcomes.

**Table 2:** Clinical outcomes of normally divided blastocysts grouped on basis of their morphological quality.

Blastocyst quality	Number (*n*)	Fraction (%)	PR (%)	CPR (%)	LBR (%)
Top Quality Blastocysts	1,461	50.9	58.3^a^	50.5^a^	38.0^a^
Good Quality Blastocysts	1,205	41.8	48.8^b^	41.1^b^	32.3^b^
Low Quality Blastocysts	203	7.1	36.9^c^	27.0^c^	22.8^c^
*p*-value			<0.001	<0.001	<0.001

Data are shown as numbers and fractions, and Fisher exact test was used to evaluate the differences between proportions. Superscript letters (a, b, c) indicate significant differences between groups; values that do not share a letter differ significantly.

PR, pregnancy rate; CPR, clinical pregnancy rate; LBR, live birth rate; all expressed per transfer.

### 3.3 Abnormal cell divisions, morula compaction and outcomes

#### 3.3.1 Errors in the first mitosis

Abnormal cleavage in the first mitosis was observed in 95 transferred blastocysts (3.1%).

Among the DUC1-3 embryos (*n* = 11), 91% developed into PCM and 9% into FCM, and most formed GQB (*n* = 5, 45%), LQB (*n* = 4, 36%) with only one blastocyst developing into TQB (9%). The eleven DUC1-3 were transferred contrary to clinical protocol; none resulted in a live birth.

All RaC embryos (*n* = 83) developed into PCM and formed clinical useful blastocysts on day 5 (57.1%) and day 6 (42.9%), developing significantly more slowly than embryos with normal cleavage (*p* < .001). A larger proportion of these blastocysts were of lower quality, predominately GQB (45.2%), followed by TQB (44%) and LQB (9.5%).

#### 3.3.2 Errors in the second mitosis

Abnormal cleavage in second mitosis was observed in 137 transferred blastocysts (4.4%), categorizing them as DUC2–5 embryos. DUC2–5 embryos were significantly less likely to form FCM than normally dividing embryos (2.1% vs. 63.8%, *p* < .001). They also developed more slowly as more of them were vitrified on day 6 (32.8% vs. 15.6%, *p* < .001). Additionally, they were more likely to develop into LQB (22.6% vs. 7.0%, *p* < .001).

Because DUC2–5 and RaC embryos displayed similar outcomes with respect to morula compaction patterns and morphological quality, and because both may contain mixtures of presumptive euploid and aneuploid cells resulting from division errors, we combined the two groups, hereafter referred to as Abnormal Cleavage (AC) and compared the clinical outcomes of AC-PCM (*n* = 227) with NC-PCM (*n* = 1,040). AC-PCM had significantly lower clinical outcomes than AC-FCM; PR 40.5% vs. 49%, CPR 30.4% vs. 40.8% and LBR 24.7% vs. 30.5%, (*p* < .01).

### 3.4 Clinical outcomes combining division patterns, morula compaction and blastocyst quality and multivariate models

Clinical outcomes differed markedly when blastocyst morphology, morula compaction pattern, and division pattern were considered together (Table 3). Among top-quality blastocysts, those originating from normally cleaved, fully compacted morulae (NC-FCM) had the highest PR, CPR and LBR, whereas AC-PCM blastocysts showed significantly lower outcomes (all *p* < .001). A similar pattern was observed for good-quality blastocysts, where AC-PCM again showed the lowest LBR and differed significantly from both NC-FCM and NC-PCM (*p* < .05). In contrast, among low-quality blastocysts, clinical outcomes did not differ significantly across division or compaction groups (all p > .05).

**Table 3:** Clinical outcomes for transferred blastocysts combining morphology quality, morula compaction pattern and division pattern.

Top Quality Blastocysts	Number (*n*)	PR (%)	CPR (%)	LBR (%)
Normal Cleavage-Fully Compacted Morula	1,098	59.2^a^	51.5^a^	38.9
Normal Cleavage-Partial Compacted Morula	363	55.8^a^	47.6^a^	35.4
Abnormal Cleavage-Partial Compacted Morula	82	47.6^b^	34.1^b^	28.0
*p*-value		<0.001	<0.001	<0.001
Good Quality Blastocysts				
Normal Cleavage-Fully Compacted Morula	642	49.8	42.3	34.6^a^
Normal Cleavage-Partial Compacted Morula	563	47.6	39.9	29.6^a^
Abnormal Cleavage-Partial Compacted Morula	102	40.2	30.4	23.5^b^
*p*-value		0.337	0.074	<0.05
Low Quality Blastocysts				
Normal Cleavage-Fully Compacted Morula	87	41.4	32.2	27.1
Normal Cleavage-Partial Compacted Morula	116	33.6	23.3	19.6
Abnormal Cleavage-Partial Compacted Morula	41	29.3	24.4	22
*p*-value		0.638	0.538	0.539

Data are shown as numbers and fractions. Differences between proportions were evaluated using chi-square test or Fisher's exact test where appropriate. Superscript letters (a, b) indicate significant differences between groups; values that do not share a letter differ significantly.

PR, pregnancy rate; CPR, clinical pregnancy rate; LBR, live birth rate; all expressed per transfer.

Overall, embryos exhibiting abnormal cleavage followed by partial compaction consistently showed poorer outcomes within each morphology grade, with the largest differences observed in top- and good-quality embryos.

An adjusted analysis accounting for maternal age and blastocyst day is presented in Supplementary Table S1, confirming the overall hierarchy of outcomes across the combined morphology–compaction–division groups.

#### 3.4.1 Multivariate analysis

**Figure 2:**
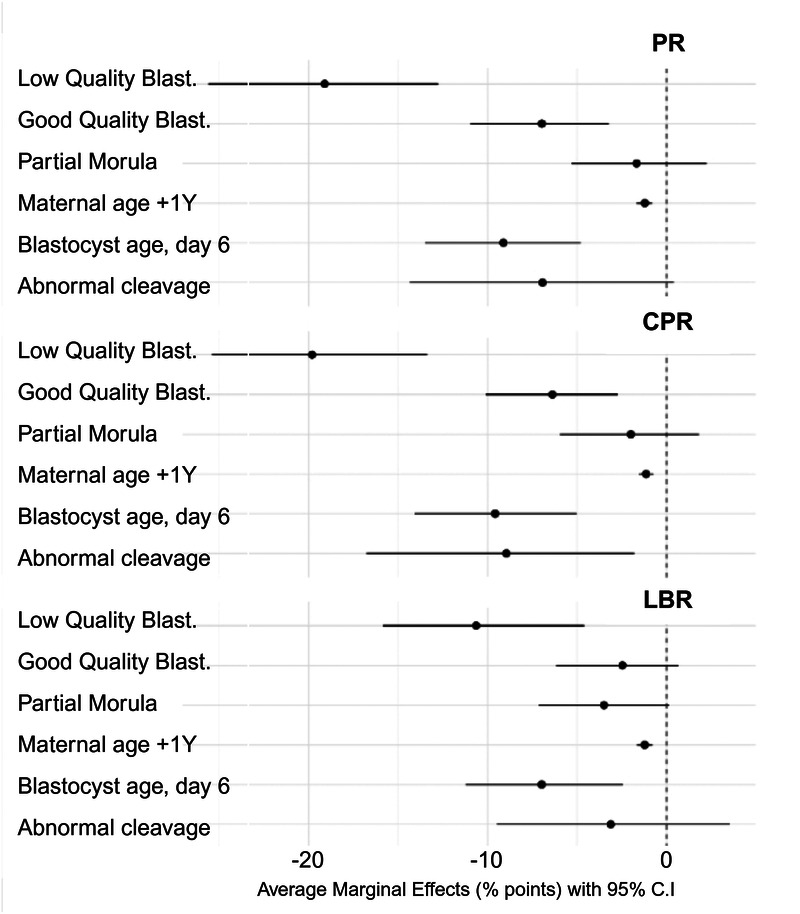
Forest plot of predictors of live birth. Average marginal effects (AME) with 95% confidence intervals for predictors of pregnancy rate (PR), clinical pregnancy rate (CPR), and live birth rate (LBR). Each row represents a predictor level compared with its reference category. Negative AME values indicate reduced probability of the outcome. Morphological quality (Good Quality Blastocysts, Low Quality Blastocysts) and blastocyst age (day 6 vs. day 5) consistently showed the largest negative effects across all outcomes. Created in BioRender. Adolfsson, E. (2026) https://BioRender.com/9a35zzb.

In the multivariable analyses, several embryo-level characteristics remained independently associated with pregnancy outcomes (Tables 4–6, Figure 2). For PR, low morphological quality showed the strongest negative effect (AME −19.1 pp), followed by Day 6 blastocysts (–9.1 pp) and abnormal cleavage (–6.9 pp). Good quality blastocysts demonstrated a smaller but significant reduction (–7.0 pp), while partial compaction had no meaningful effect. Maternal age showed a consistent decrease of −1.2 percentage points per year.

**Table 4:** Average marginal effects (AME) with 95% confidence intervals for predictors of pregnancy rate (PR).

Predictor	Comparison	AME (% points)	C.I	*p*-value
Low Quality Blastocyst	Top Quality Blastocyst	−19.10	−25.60 to −12.76	<0.001
Day 6 blastocyst	Day 5 blastocyst	−9.12	−13.50 to −4.80	<0.001
Good Quality Blastocyst	Top Quality Blastocyst	−6.97	−10.95 to −3.24	<0.001
Abnormal Cleavage	Normal Cleavage	−6.93	−14.34 to −0.40	0.056
Maternal age (+1 year)	Per year increase	−1.22	−1.68 to −0.82	<0.001
Partial Compaction Morula	Fully Compacted Morula	−1.68	−5.30 to −2.24	0.392

Negative AME values indicate reduced probability of pregnancy relative to the reference level for each predictor. Low quality blastocysts and day 6 blastocysts showed the largest reductions in PR.

**Table 5:** Average marginal effects (AME) with 95% confidence intervals for predictors of clinical pregnancy rate (CPR).

Predictor	Comparison	AME (% points)	C.I (95%)	*p*-value
Low Quality Blastocyst	Top Quality Blastocyst	−19.8	−25.4 to −13.38	<0.001
Day 6 blastocyst	Day 5 blastocyst	−9.58	−14.08 to −5.05	<0.001
Abnormal Cleavage	Normal Cleavage	−8.95	−16.75 to −1.8	<0.05
Good Quality Blastocyst	Top Quality Blastocyst	−6.38	−10.11 to −2.72	<0.001
Maternal age (+1 year)	Per year increase	−1.15	−1.55 to −0.72	<0.001
Partial Compaction Morula	Fully Compacted Morula	−2.0	−5.95 to 1.81	0.330

The pattern of effects closely mirrors the PR model (Table 4), with low-quality morphology and day 6 blastocysts showing the strongest negative associations.

**Table 6:** Average marginal effects (AME) with 95% confidence intervals for predictors of live birth rate (LBR).

Predictor	Comparison	AME (% points)	C.I (95%)	*p*-value
Low quality blastocyst	Top quality	−10.64	−15.85 to −4.61	<0.001
Day 6 blastocyst	Day 5	−6.98	−11.23 to −2.47	<0.05
Maternal age (+1 year)	Per year increase	−1.23	−1.67 to −0.81	<0.001
Partial compaction	Full compaction	−3.5	−7.14 to 0.13	0.063
Abnormal cleavage	Normal cleavage	−3.12	−9.48 to 3.5	0.362
Good quality blastocyst	Top quality	−2.47	−6.16 to 0.67	0.162

Low morphological quality and day 6 blastocysts remained significant predictors after adjustment, although effect sizes were smaller than for PR (Table 4) and CPR (Table 5).

The pattern was similar for CPR, where low quality blastocysts remained the strongest predictor (–19.8 pp). Day 6 blastocysts (–9.6 pp) and abnormal cleavage (–8.9 pp) also showed significant reductions, whereas partial compaction again demonstrated no clear effect. Good-quality blastocysts showed a moderate reduction (–6.4 pp), and maternal age was again associated with a decline of approximately −1.2 points per year.

For LBR, low morphological quality continued to show the largest adjusted effect (–10.6 pp). Day 6 blastocysts were associated with reduced live-birth probability (–7.0 pp). Abnormal cleavage (–3.1 pp) and partial compaction (–3.5 pp) showed smaller and statistically uncertain effects, while good-quality blastocysts demonstrated a modest reduction (–2.5 pp). As with PR and CPR, maternal age remained a stable negative predictor.

Together, these findings indicate that morphological grade and blastocyst developmental timing exert the largest independent effects on embryo competence, with abnormal cleavage showing a consistent influence on PR and CPR but not LBR.

A complementary likelihood-ratio test analysis confirmed that partial compaction status was not significantly associated with pregnancy, clinical pregnancy, or live birth (all LRT *p* > .30, *Δ*AIC <2), whereas abnormal cleavage was associated with reduced pregnancy and clinical pregnancy rates, although its effect did not persist at the live-birth stage.

## 4 Discussion

This large retrospective time-lapse study re-evaluated more than 3,100 transferred blastocysts with known outcomes, integrating early division patterns, morula compaction and subsequent morphological quality. Most transferred blastocysts originated from normally cleaving embryos (NC), and within the NC group, the largest subgroup consisted of fully compacted morulae (NC-FCM). Across analyses, top-quality NC-FCM blastocysts achieved the highest clinical success (LBR 38.9% per transfer). In multivariable models, morphological grade and blastocyst developmental day emerged as the strongest independent predictors of outcome, maternal age showed a consistent negative association (∼1 percentage point per year), abnormal cleavage was associated with reduced PR/CPR but not LBR, and partial compaction did not retain significance after adjustment. Taken together, these results indicate that morphological grade and blastocyst developmental timing are the strongest independent predictors of live-birth probability, while cleavage-stage abnormalities primarily affect earlier endpoints (PR/CPR). Although morphology and blastocyst day are well-established predictors, our study uniquely integrates early cleavage behaviour, morula compaction, and final morphology. The sample size of the present study provides a level of statistical resolution that exceeds previous studies.

In the present study, the NC rate was higher than that reported by Parriego et al. (23) who observed 74.8% NC with a FCM rate of 50.7%, and by De Martin et al. (1), who reported 82% NC with similar FCM rate of 60%. Notably, patients in the present study were substantially younger—on average by almost six years—which may explain the difference. Although Parriego et al. and de Martin et al. did not observe a correlation between female age and abnormal cell-division frequency or morula compaction pattern it is plausible that the accumulation of meiotic errors with advancing maternal age contributes to abnormal cell divisions (27), potentially explaining the lower incidence in our cohort. It is also likely that awareness of abnormal cell divisions has influenced embryo selection, with embryos without deviations consistently prioritized when multiple blastocysts are available. The high clinical success in this cohort, with a cumulative LBR of 65.1%, also indicates that our patients represents a good-prognosis population. Compared with prior studies, the substantially larger number of transferred blastocysts in our study allows a more precise estimation of how cleavage and compaction patterns translate into clinical outcomes.

One hypothesis commonly embraced by embryologists is that “all sins are forgiven” if the embryo reaches the blastocyst stage. This notion is partially supported in the literature, which describes self-correction mechanisms during compaction including exclusion or extrusion of aberrant cells (28, 29), and reports that PCM blastocysts can be euploid at rates comparable to, or even exceeding, those of FCM (15). Consistent with this framework, we observed that AC embryos predominantly developed into PCM, and that within each morphology category, NC-FCM outperformed both NC-PCM and AC-PCM in unadjusted comparisons. Further studies are warranted to determine why outcomes differed between NC-PCM and AC-FCM. One plausible biological explanation is that that a larger proportion of cells were excluded in AC-PCM morulae than in NC-PCM morulae, reflecting the need to eliminate all presumably aneuploid cells arising from abnormal cleavage (RaC and/or DUC2-5). Future studies could apply the scoring criteria proposed by Fabozzi et al. to separate PCM into four morphological classes, to further examine the scoring potential of morula morphology and compaction pattern beyond the binary system used in the present study (30). Our findings complement this hypothesis by showing that even when self-correction is possible, cleavage-stage abnormalities still left a measurable imprint on implantation potential in large real-world transfer cohorts.

Despite the potential for euploidy in PCM, outcomes remained inferior to FCM across morphology strata. Previous work has linked reduced cell allocation or embryo mass at the morula-to-blastocyst transition with diminished developmental competence (30, 31). Our findings align with this literature, showing that optimal cleavage, full compaction, and superior morphology together yield the highest clinical success. Importantly, our study links compaction pattern to live-birth outcomes in a much larger set of single-blastocyst transfers than previously reported, strengthening the clinical relevance of these associations.

Only 11 transferred blastocysts with DUC1–3 were identified; none resulted in live birth which is consistent with prior reports (5, 29). Retrospective time-lapse analyses inherently risk reflecting historical selection deviations as knowledge evolves; such transfers would not meet current SOPs. DUC2–5 and RaC embryos were almost exclusively PCM, reinforcing the link between early cleavage errors, compaction behaviour and downstream competence. In contrast, reverse cleavage was not classified as an abnormal event in this study consistent with the findings of Jin et al. (11). Our small reverse-cleavage subset (*n* = 32) yielded seven live births, consistent with evidence that blastulation reverse-cleavage embryos can have euploidy rates comparable to normally dividing cells (11). Although numbers are limited, these observations support that reverse cleavage *per se* is not an exclusion criterion when subsequent development is robust. These data provide rare real-world outcome information for transfer decisions involving embryos with uncommon cleavage abnormalities, an area where existing literature is sparse.

Our results differ from Coticchio et al (17)., who identified compaction pattern as an independent predictor of live birth after multivariable adjustment. This divergence may reflect differences in study design, cohort characteristics, timing and criteria of compaction assessment, or analytical approaches. In our dataset, compaction pattern did not retain significance once morphological grade and blastocyst day—both stronger predictors—were included in the model. By adjusting for morphology and blastocyst day in a much larger clinical cohort, our study suggests that the independent predictive value of compaction may depend on model specification, and that accounting for morphology and blastocyst day alters the apparent strength of this association.

Notably, morphological grade remained the dominant predictor across PR, CPR and LBR, even after accounting for maternal age, cleavage pattern and compaction. This underscores the continued central importance of morphology in embryo selection. Time-lapse derived parameters (cleavage patterns, compaction behaviour) provided complementary information, particularly evident for AC effects on PR/CPR, while the primary predictive weight remained in morphology and developmental timing.

Strengths include the large cohort of individually transferred blastocysts with clinical outcomes and blinded re-annotation of time-lapse sequences with morphology included, reflecting real-world selection practices. To our knowledge, this is the largest study on morula compaction patterns. Limitations stem from the retrospective single-centre design spanning more than a decade; although culture conditions were stable, unmeasured factors (e.g., staffing, patient demographics, stimulation regimes) may have varied. Because only transferable blastocysts were analyzed, the frequency of abnormal divisions is likely under-represented relative to the full embryo population (29), resulting in a much larger NC than AC category. Additional developmental features known to affect competence such as unequal blastomere size, severe fragmentation, nuclear anomalies or blastocyst collapses were not incorporated (3, 32, 33). Finally, implantation may fail despite embryo competence due to factors such as endometrial receptivity and other maternal factors, which were beyond the scope of this study. As this is a single-centre study our findings need to be confirmed in multi-centre settings. Despite these limitations, our findings add to the growing evidence that errors in the first two mitosis are important to capture to improve embryo selection. Culture to the blastocyst stage and observation of the morula stage may aid in identifying embryos with the highest likelihood of resulting in live birth after assisted reproduction.

These data support integrating early developmental information—cleavage pattern and compaction—with morphology and blastocyst day when evaluating embryos. Within each morphology grade, embryos without abnormal cleavage and with full compaction consistently achieved superior outcomes. Overall, early mitotic errors and compaction behaviour carry measurable consequences for embryo competence, while morphological grade and developmental timing remain the principal determinants of live birth.

## Data Availability

The raw data supporting the conclusions of this article will be made available by the authors, without undue reservation.
