# Supplementary material for: Cleavage pattern, morula compaction and blastocyst morphology as determinants of live birth after single blastocyst transfer
Source: Front Reprod Health. 2026 Apr 15;8:1822869. doi: 10.3389/frph.2026.1822869 (PMC13124717; doi:10.3389/frph.2026.1822869)
Supplement: Supplementary file 1 [file Table1.docx]

Supplementary Material

# Supplementary Data

### S1 Video

Direct Unequal Cleavage at the first mitosis (DUC1-3) resulted in three daughter cells. Each daughter cell divided further.

### S2 Video

Direct Unequal Cleavage at the second mitosis (DUC2-5) resulted in five daughter cells from the two cells. Each daughter cell divided further.

### S3 Video

Rapid Cleavage at the first mitosis (RaC) resulted in three cells. First, normal mitosis formed two cells. One of these rapidly, within five hours, cleaved again, resulting in a three-cell embryo. Each daughter cell divided further.

### S4 Video

Formation of a Fully Compacted Morula (FCM) with inclusion of all mitotic cells.

### S5 Video

Formation of a Partial Compacted Morula (PCM) where cells are excluded from the forming morula.

# Supplementary Tables

**Supplemental Table 1. Adjusted odds ratio with 95 % confidence interval in relation to top quality blastocysts originating from normally cleaved embryos forming fully compacted morulas.** Groups are presented in descending order based on live birth rate. Odds ratios are adjusted for maternal age and blastocyst age.

| Category | Number | LBR (%) | Odds Ratio (C.I.) | p-value |
| --- | --- | --- | --- | --- |
| Top Quality Blastocyst, Fully Compacted, Normal Cleavage | 1098 | 38.9 | 1.0 (reference) | - |
| Top Quality Blastocyst, Partial Morula, Normal Cleavage | 363 | 35.4 | 0.87 (0.68-1.12) | n.s. |
| Good Quality Blastocyst, Fully Compacted, Normal Cleavage | 642 | 34.6 | 0.88 (0.72-1.08) | n.s. |
| Good Quality Blastocyst, Partial Morula, Normal Cleavage | 563 | 29.6 | 0.67 (0.54-0.84) | p<0.001 |
| Top Quality Blastocyst, Partial Morula, Abnormal Cleavage | 82 | 28.0 | 0.68 (0.41-1.09) | n.s. |
| Low Quality Blastocyst, Fully Compacted, Normal Cleavage | 87 | 27.1 | 0.63 (0.38-1.00) | n.s |
| Good Quality Blastocyst, Partial Morula, Abnormal Cleavage | 102 | 23.5 | 0.54 (0.33-0.85) | P<0.01 |
| Low Quality Blastocyst, Partial Morula, Abnormal Cleavage | 41 | 22.0 | 0.49 (0.22-0.99) | n.s. |
| Low Quality Blastocyst, Partial Morula, Normal Cleavage | 116 | 19.6 | 0.41 (0.25-0.65) | p<0.001 |
